# The Effect of Diabetes Mellitus Type 1 on the Energy Metabolism of Hepatocytes: Multiphoton Microscopy and Fluorescence Lifetime Imaging

**DOI:** 10.3390/ijms242317016

**Published:** 2023-11-30

**Authors:** Svetlana Rodimova, Nikolai Bobrov, Artem Mozherov, Vadim Elagin, Maria Karabut, Polina Ermakova, Ilya Shchechkin, Dmitry Kozlov, Dmitry Krylov, Alena Gavrina, Aleksandra Kashina, Vladimir Zagainov, Elena Zagaynova, Daria Kuznetsova

**Affiliations:** 1 Institute of Experimental Oncology and Biomedical Technologies, Privolzhsky Research Medical University, 10/1 Minin and Pozharsky Sq., 603000 Nizhny Novgorod, Russia; srodimova123@gmail.com (S.R.); elagin.vadim@gmail.com (V.E.); mail-kozlov2015@yandex.ru (D.K.); dmitr.krilow2013@yandex.ru (D.K.);; 2 The Volga District Medical Centre of Federal Medical and Biological Agency, 14 Ilinskaya St., 603000 Nizhny Novgorod, Russia; 3 Laboratory of Molecular Genetic Research of the Institute of Clinical Medicine, Lobachevsky Nizhny Novgorod National Research State University, 23 Gagarina Ave., 603022 Nizhny Novgorod, Russia; 4 Nizhny Novgorod Regional Clinical Oncologic Dispensary, 11/1 Delovaya St., 603126 Nizhny Novgorod, Russia; 5 Lopukhin Federal Research and Clinical Center of Physical-Chemical Medicine of Federal Medical Biological Agency, 1a Malaya Pirogovskaya St., 119435 Moscow, Russia

**Keywords:** multiphoton microscopy, FLIM, SHG, liver regeneration, diabetes mellitus

## Abstract

A decrease in the regenerative potential of the liver during the development of non-alcoholic fatty liver disease (NAFLD), which is observed in the vast majority of patients with diabetes mellitus type 1, significantly increases the risk of postoperative liver failure. In this regard, it is necessary to develop new approaches for the rapid intraoperative assessment of the condition of liver tissue in the presence of concomitant liver pathology. A modern label-free approach based on multiphoton microscopy, second harmonic generation (SHG), and fluorescence lifetime imaging microscopy (FLIM) allow for the evaluation of the structure of liver tissue as well as the assessment of the metabolic state of hepatocytes, even at the cellular level. We obtained optical criteria and identified specific changes in the metabolic state of hepatocytes for a reduced liver regenerative potential in the presence of induced diabetes mellitus type 1. The obtained criteria will expand the possibilities for the express assessment of the structural and functional state of liver tissue in clinical practice.

## 1. Introduction

Until now, the only treatment option for patients with primary and secondary liver cancers is liver resection, but the 5-year survival rate is only 14–61% [1,2]. Despite modern advances in methods for the preoperative and intraoperative assessment of liver structure and function, there is currently a high risk of postoperative liver failure associated with the presence of background hepatic and systemic pathologies. One of these diseases that significantly reduces liver regenerative potential is diabetes mellitus type 1. The World Health Organization predicts that 300 million people around the world will suffer from diabetes mellitus (DM) by 2025. DM is a systemic metabolic disorder characterized by hyperglycemia, hyperlipidemia, and organismic insulin resistance [3,4]. It alters the carbohydrate, lipid, and protein metabolism of patients. In some cases, DM causes the excessive accumulation of lipids in the liver, resulting in a fatty liver and, consequently, nonalcoholic fatty liver disease (NAFLD). Subsequently, 2–3% of NAFLD patients experience hepatic inflammation, necrosis, and fibrosis, which are symptoms of a condition known as non-alcoholic steatohepatitis (NASH). This can lead to patients over 45 with DM, obesity, and dyslipidemia having a higher chance of, at a minimum, stage 2 fibrosis [5,6,7,8]. Patients with NAFLD or NASH show a higher incidence of complications and mortality after liver surgery [9].

DM is considered a risk factor affecting prognosis after liver resection in patients with hepatocellular carcinoma. Post-surgery mortality is higher in diabetic patients, as DM increases the probability of liver decompensation. In patients subjected to a major hepatectomy, DM tends to induce postoperative liver failure [10]. The post-surgical recovery mass depends on the regenerative ability of the remnant liver. Several studies suggest that insulin deficiency leads to distorted intracellular signaling pathways in both type 1 [11] and type 2 [8,12] diabetic livers and, as a result, the regenerative response is deficient as [11] DM generally involves increased free radical production [7,13,14,15] or reduced antioxidant defense [16,17]. An increase in lipid peroxidation was found to be important for normal proliferative processes to occur in the liver remnant after partial hepatectomy (PH), but this could result in DNA damage. Oxygen free radicals are extensively formed in diabetic patients by glucose oxidation, non-enzymatic protein glycation and subsequent oxidative degradation of the glycated proteins [13]. In addition, pathological shifts in both the circulating fuel levels and energy substrate utilization by central and peripheral tissues contribute to mitochondrial dysfunction across multiple organ systems. Mitochondria lie at the intersection of critical cellular pathways such as energy substrate metabolism, reactive oxygen species (ROS) generation, and apoptosis. It is the disequilibrium of these processes in DM that results in downstream deficits in vital functions, including hepatocyte metabolism [4].

Approaches based on fluorescence bioimaging methods, such as multiphoton microscopy, second harmonic generation (SHG) microscopy, and fluorescence lifetime imaging microscopy (FLIM) are expanding the possibilities of biomedical research. These methods are label-free and minimally invasive, allowing analysis of not only the structure of the liver, but also the metabolic state of the hepatocytes. Using FLIM, we can assess changes in the metabolic state of the hepatocytes based on data on the fluorescence lifetimes and relative contributions of various forms of the intracellular cofactors NAD(P)H. The free form of NADH is localized in the cytosol and involved in glycolysis; its bound form is localized in mitochondria and involved in the tricarboxylic acid cycle and oxidative phosphorylation (OXPHOS), while NADPH (the phosphorylated form of NADH) is involved in biosynthetic processes, primarily in lipid metabolism and antioxidant protection.

Previously, we have confirmed the effectiveness of this approach for the express assessment of liver tissue, and we have also identified criteria indicating reduced liver regenerative potential at different stages of steatosis and fibrosis [18]. The purpose of this current work was to identify criteria associated with a reduction of the liver regenerative potential in the presence of a systemic complex disease—DM, which inevitably leads to severe fatty liver disease.

## 2. Results

### 2.1. Blood Glucose Assessment

The induction of diabetes mellitus type 1 was confirmed by measuring blood glucose levels. The average blood glucose level in rats with STZ-induced (45 mg/kg dose) DM was 28.4 ± 4.5 mmol/L. DM remained stable for 90 days. The results of blood glucose monitoring in the rats are shown in Figure S1.

The development of DM in rats was also confirmed by a glucose tolerance test. We showed that in rats with DM, the level of glucose in the blood did not undergo any increase in the first 20 min after the administration of 40% glucose, while in healthy rats there was an increase by an average of 6–13 mmol/L. An hour later, in the healthy animals, the glucose level had then fallen by 10 mmol/L, while in animals with STZ-induced DM, the glucose level either did not decrease or decreased by only 2–3 mmol/L.

### 2.2. Multiphoton Microscopy and SHG

**Figure 1:**
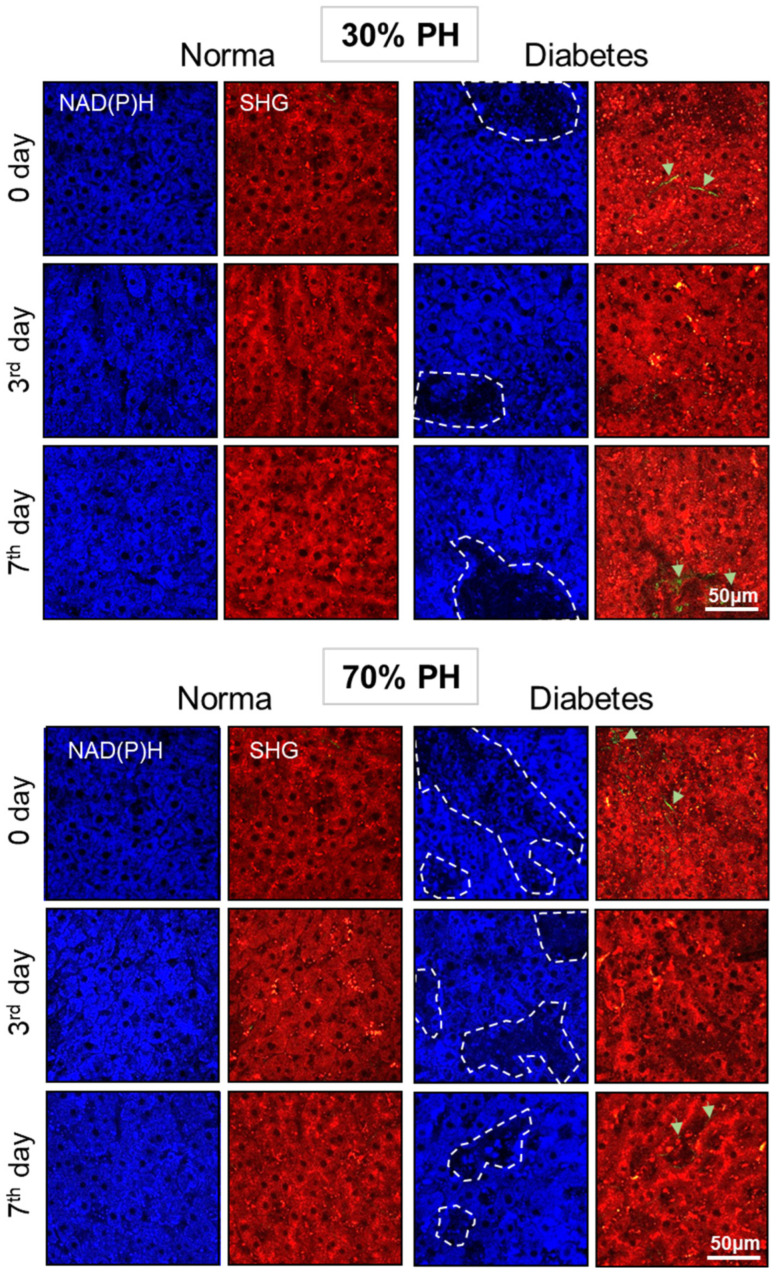
Fluorescence intensity images of NAD(P)H autofluorescence; NAD(P)H: excitation at 750 nm, detection range 455–500 nm; white dotted lines indicate areas with reduced NAD(P)H autofluorescence intensity. Cell autofluorescence (red): excitation at 800 nm, detection range 433–660 nm, SHG of collagen (green): excitation at 800 nm, detection range 371–421 nm; green arrows indicate collagen. Normal regeneration—30% PH (*n* = 20, for day 0, and *n* = 10, for the 3rd and 7th days) and 70% PH (*n* = 20, for day 0, and *n* = 10, for the 3rd and 7th days), DM—30% PH (*n* = 12, for day 0, and *n* = 6, for the 3rd and 7th days) and 70% PH (*n* = 12, for day 0, and *n* = 6, for the 3rd and 7th days).

Using multiphoton microscopy, we analyzed the structure of the liver tissue. Also, using SHG, we analyzed the accumulation of collagen in the liver tissue during the development of DM. In liver tissue undergoing normal regeneration, the NAD(P)H autofluorescence signal was uniformly distributed with a small number of bright inclusions (signals from vitamin A) (Figure 1) [19]. In addition, the SHG analysis showed there was no accumulation of collagen during normal liver regeneration.

In the liver tissue with concomitant DM, we revealed the appearance of zones with a reduced NAD(P)H autofluorescence signal that were associated either with areas of lipid infiltration or with the foci of fibrosis. Also, individual foci of fibrosis were observed. Thus, it was shown that specific optical criteria for the presence of pathological changes in the liver in concomitant DM are the appearance of zones with a reduced NAD(P)H autofluorescence signal associated with the foci of lipid infiltration and the foci of fibrosis (Figure 1).

**Figure 2:**
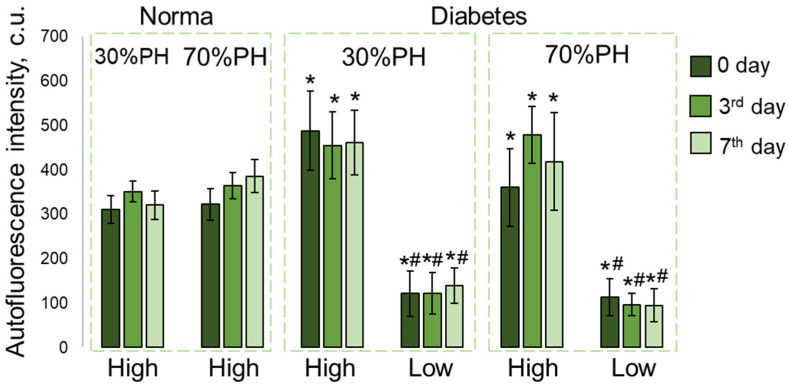
Analysis of the NAD(P)H autofluorescence intensity in liver tissue during normal regeneration and during regeneration with induced DM. The analysis was carried out in two identified zones: with high and low NAD(P)H autofluorescence intensity. *—statistically significant differences compared to the corresponding time point for normal regeneration, #—statistically significant differences compared to the corresponding time point of this corresponding PH model for the area with a high NAD(P)H autofluorescence intensity; *p*-value ≤ 0.05. Normal regeneration—30% PH (*n* = 20, for day 0, and *n* = 10, for the 3rd and 7th days) and 70% PH (*n* = 20, for day 0, and *n* = 10, for the 3rd and 7th days), DM—30% PH (*n* = 12, for day 0, and *n* = 6, for the 3rd and 7th days) and 70% PH (*n* = 12, for day 0, and *n* = 6, for the 3rd and 7th days).

A quantitative assessment of the autofluorescence signal intensity in the NAD(P)H channel was carried out in two identified zones, those with low and high NAD(P)H autofluorescence signals. During normal regeneration, both for 30% PH and for 70% PH, the NAD(P)H autofluorescence signal was uniform, and no zones with a reduced signal were observed. However, in pathology, in zones with a high intensity of NAD(P)H autofluorescence, the intensity was significantly higher in comparison with normal regeneration for both 30% PH and for 70% PH. In zones with a low NAD(P)H autofluorescence signal, the intensity was significantly reduced, both in comparison with normal regeneration and with zones with a high NAD(P)H autofluorescence signal in DM. The results of this quantitative assessment of the intensity of the NAD(P)H autofluorescence signal in liver tissue during regeneration in DM are presented in Figure 2.

### 2.3. FLIM Analysis

Using FLIM, we revealed changes in the metabolic state of hepatocytes during normal regeneration and during regeneration with concomitant DM. We analyzed the fluorescence lifetimes of the bound form of NADH (t2, ps), NADPH (t3, ps), and the amplitude weighted mean fluorescence lifetimes (tm, ps). For the 30% PH model, a significant decrease in the values of tm was shown with concomitant DM, in comparison with the corresponding time points of normal regeneration after 30% PH. For the 70% PH model, a significant decrease in the values of t1 and tm were shown with concomitant DM, in comparison with the corresponding time points of normal regeneration after 70% PH. Such a decrease in the t1 and tm parameters probably indicates hypoxia in hepatocytes. Also, for the 70% PH model, a significant increase in t3 was shown, which indicates an increase in antioxidant defense in hepatocytes [20]. Full data are presented in Table S1.

**Figure 3:**
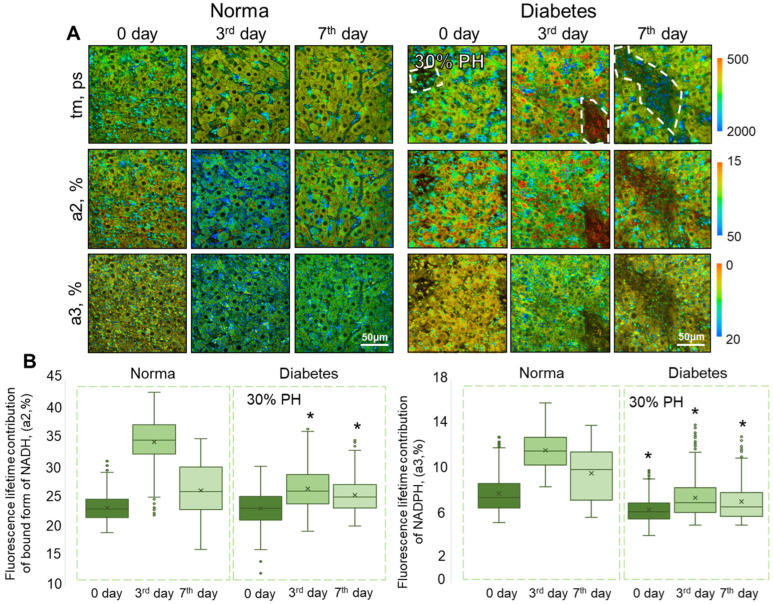
FLIM analysis of liver tissue during normal liver regeneration and during liver regeneration with induced DM. (**A**) Pseudo-coded FLIM images of normal and diabetic livers before 30% PH (day 0) and on the 3rd and 7th days after 30% PH; scale bar 50 μm; ×400. (**B**) Boxplots reflecting the distribution of the values of the fluorescence lifetime contributions of the bound form of NADH and NADPH. *—statistically significant differences compared to the corresponding time point for normal regeneration, *p*-value ≤ 0.05. Normal regeneration—30% PH (*n* = 20, for day 0, and *n* = 10, for the 3rd and 7th days) and 70% PH (*n* = 20, for day 0, and *n* = 10, for the 3rd and 7th days), DM—30% PH (*n* = 12, for day 0, and *n* = 6, for the 3rd and 7th days) and 70% PH (*n* = 12, for day 0, and *n* = 6, for the 3rd and 7th days).

**Figure 4:**
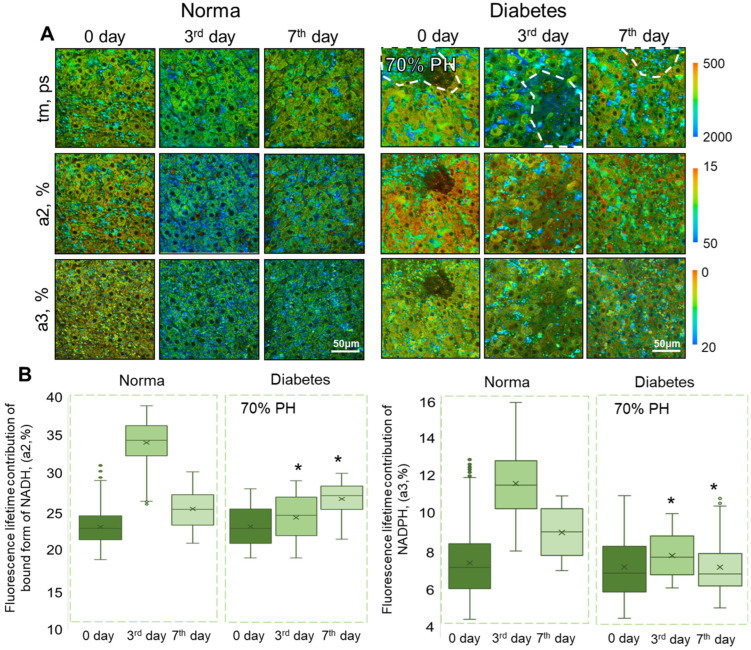
FLIM analysis of liver tissue during normal liver regeneration and during liver regeneration with induced DM. (**A**) Pseudo-coded FLIM images of normal and diabetic livers before 70% PH (day 0) and on the 3rd and 7th days after 70% PH; scale bar 50 μm; ×400. (**B**) Boxplots reflecting the distribution of the values of the fluorescence lifetime contributions of the bound form of NADH and NADPH. *—statistically significant differences compared to the corresponding time point for normal regeneration, *p*-value ≤ 0.05. Normal regeneration—30% PH (*n* = 20, for day 0, and *n* = 10, for the 3rd and 7th days) and 70% PH (*n* = 20, for day 0, and *n* = 10, for the 3rd and 7th days), DM—30% PH (*n* = 12, for day 0, and *n* = 6, for the 3rd and 7th days) and 70% PH (*n* = 12, for day 0, and *n* = 6, for the 3rd and 7th days).

It was shown that on the 3rd day of normal regeneration, for both 30% PH and 70% PH, there was a sharp increase in the values of the contributions of the fluorescence lifetime of the bound form of NADH (a2, %) and NADPH (a3, %) in the hepatocytes, compared with day 0 (before PH). On the 7th day there was a significant decrease in the values of a2 and a3, although not dropping as low as the initial values (before PH) (Figure 3 and Figure 4).

In the 30% PH model, in the presence of concomitant DM, the a2 values on day 0 did not differ from the normal values. By contrast, the values of a3 on day 0 in the diabetic liver were already reduced compared to normal liver. On the 3rd day of regeneration in pathology, no sharp increase in the values of either a2 or a3 had occurred. By the 7th day of liver regeneration in pathology, the values of a2 and a3 still did not differ significantly from days 0 and 3 after PH.

In the 70% PH model, there was similarly no sharp jump in the values of a2 and a3 on the 3rd day of diabetic liver regeneration. However, in contrast to the 30% PH situation, by the 7th day of regeneration following 70% PH, a significant increase in the values of a2 occurred. This result probably indicates delayed metabolic changes associated with liver regeneration in DM. It is also consistent with the data from morphometric analysis, which showed the activation of compensatory proliferation of hepatocytes, due to the resection of large volumes of liver. Interestingly, the values of a3 did not change significantly at any of the monitoring time points during diabetic liver regeneration.

### 2.4. Real-Time PCR

Firstly, we carried out a correlation analysis in order to determine the significance of the factors such as a specific day of regeneration and/or the presence of DM. For the 30% PH model, a significant effect of the interaction between the “day” and “pathology” factors was found for the genes *Il6ra* (*p* < 0.05), *Nr1h3* (*p* < 0.01), *Ppara* (*p* = 0.065), *Cyp7a1* (*p* = 0.070), and *Ucp2* (*p* = 0.085); for the 70% PH model, a significant effect of the interaction of the “day” and “pathology” factors was found for the genes *Srebf2* (*p* < 0.01), *Ppara* (*p* < 0.001), *Nr1h3* (*p* < 0.001), *Il1a* (*p* < 0.01), *Slc2a2* (*p* < 0.01), *HGF* (*p* < 0.05), *Tnfrsf1a* (*p* < 0.05), *Cyp7a1* (*p* = 0.058), and *Ccn2* (*p* = 0.084).

The influence of the “day” factor in the 30% PH model was found for the genes *Slc2a2* (*p* < 0.001), *Acaca* (*p* = 0.057), and *Cat* (*p* = 0.068); and in the 70% PH model, it was found for the genes *Cyp2e1* (*p* < 0.001), *Ucp2* (*p* < 0.05), *Il6ra* (*p* = 0.055), Acaca (*p* < 0.05), and *Cat* (*p* = 0.056).

The influence of the “pathology” factor in the 30% PH model was found for the genes *Il6ra* (*p* < 0.01), *Mlxipl* (*p* < 0.01), *Cyp2e1* (*p* < 0.001), *Srebf1* (*p* < 0.001), *Ccn2* (*p* < 0.05), and *Tnfrsf1a* (*p* < 0.001); for the 70% PH model, it was found for the genes *Srebf2* (*p* < 0.05), *Srebf1* (*p* < 0.001), *Il1a* (*p* < 0.001), *Slc2a2* (*p* < 0.01), *Cyp2e1* (*p* < 0.001), and *Mlxipl* (*p* < 0.001). The results of the gene correlation analysis are presented in Figure S2.

**Figure 5:**
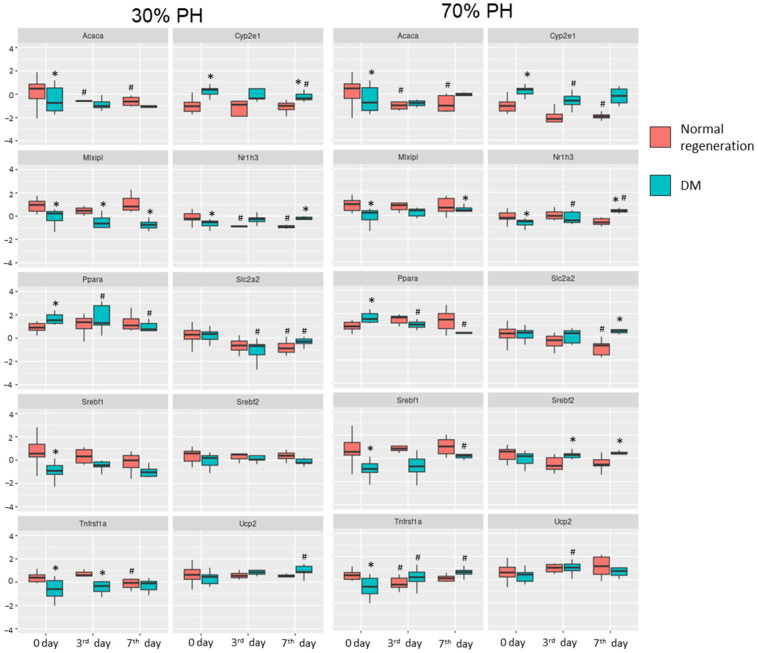
Analysis of changes in gene expression during normal regeneration and during regeneration with concomitant DM. *—statistical differences for time points of normal regeneration from the corresponding time points of regeneration with concomitant DM; *p*-value ≤ 0.05. #—statistical differences for the 3rd and 7th days from day 0 of normal regeneration or regeneration with concomitant DM; *p*-value ≤ 0.05. Normal regeneration—30% PH (*n* = 20, for day 0, and *n* = 10, for the 3rd and 7th days) and 70% PH (*n* = 20, for day 0, and *n* = 10, for the 3rd and 7th days), DM—30% PH (*n* = 12, for day 0, and *n* = 6, for the 3rd and 7th days) and 70% PH (*n* = 12, for day 0, and *n* = 6, for the 3rd and 7th days).

The results of the analysis of the expression of genes associated with liver regeneration and liver regeneration with concomitant DM are presented in Figure 5.

Under normal conditions, the insulin-driven triggering of signal cascades results in the activation of downstream targets of the PI3K—Akt path, which, together with the activation of Nr1h3, facilitates the initiation of the de novo lipogenesis process (*Mlxipl*, *Srebft1*, *Acaca*) [21,22]. Under insulin-deficient conditions (in particular, DM), the intensity of de novo lipogenesis decreases sharply, although there is a predominant uptake of TG from the blood to compensate for the decreased glucose uptake in the absence of insulin [23,24]. In general, we identified a decrease in the expression of genes associated with glucose metabolism and de novo lipogenesis, as well as demonstrating an increase in the expression of *Cyp2e1* and *Ppara* in induced DM, all these effects correlating with an increase in the expression of genes associated with antioxidant protection (such as catalase, glutathione synthase and Sirt1). Reduced expression of *Cyp7a1* also contributes to a pathological accumulation of TG in liver cells; this target gene is responsible for cholesterol catabolism and an increase in the expression of *Srebpf2*, a key activator of cholesterol synthesis. Moreover, the higher expression of *Cyp2e1* and *Ppara* in DM [25] results in an increase in ROS production, and ultimately leads to lipid oxidation and to the development of mitochondrial dysfunction. Also, we can see the activation of *Ppara* expression as a compensatory mechanism in the case of increased TG accumulation and insulin deficiency in the body this gene inhibits the transcription of *Mlxipl* (an important regulator of glucose metabolism) [26,27,28].

The development of mitochondrial dysfunction, and the decrease in the intensity of de novo lipogenesis in liver cells, is consistent with the results obtained using FLIM (the a2 and a3 values do not spike during regeneration occurring with concomitant DM). Furthermore, the pathological accumulation of TG in hepatocytes was confirmed by the results of multiphoton microscopy (increased lipid infiltration in the case of concomitant DM) and by histological analysis (the accumulation of lipid droplets in the hepatocytes).

In addition to pathological changes, we have established a decrease in the proliferative activity of the hepatocytes when there is concomitant DM. *Ppara* is known as an important hepatocyte mitogen [29,30]. This study has revealed a tendency towards an increase in *Ppara* expression during normal regeneration, compared to a pronounced decrease in such expression during regeneration with concomitant DM. A significant decrease in the expression of the *Il6ra* and *TNFR1a* proinflammatory cytokine receptors was identified in the case of concomitant DM in the 30% PH model; these being important signals for initiating regeneration. On the contrary, in the 70% PH model with concomitant DM, the expression of *Il6ra* and *TNFR1a* tended to increase, but this change is probably associated with the inflammation accompanied extensive liver damage and the development of mitochondrial dysfunction [31]. Moreover, as *TNFR1a* is an indirect antagonist of *Ppara*, this explains the decrease in *Ppara* expression in the 70% PH model with concomitant DM, which ultimately contributes to the development of hepatic steatosis. Finally, one of the most important mitogens for hepatocytes is HGF [32], its peak expression in normal regeneration occurs on the 3rd day, followed by a decrease by the 7th day. In concomitant DM, an increase in the *HGF* expression is seen only on the 7th day of regeneration. Thus, the data from this study attest to a decrease in the regenerative potential of the liver in concomitant DM, which corresponds to the results obtained using multiphoton microscopy, SHG, and FLIM.

### 2.5. Histological Analysis

During normal regeneration, the structure of the liver tissue was unchanged pathologically. On the 3rd and 7th days after 30% PH and 70% PH, transient microvesicular steatosis was observed (more than 30% of liver cells contain lipid droplets that do not displace the cell nucleus), which is characteristic of normal liver regeneration [33].

**Figure 6:**
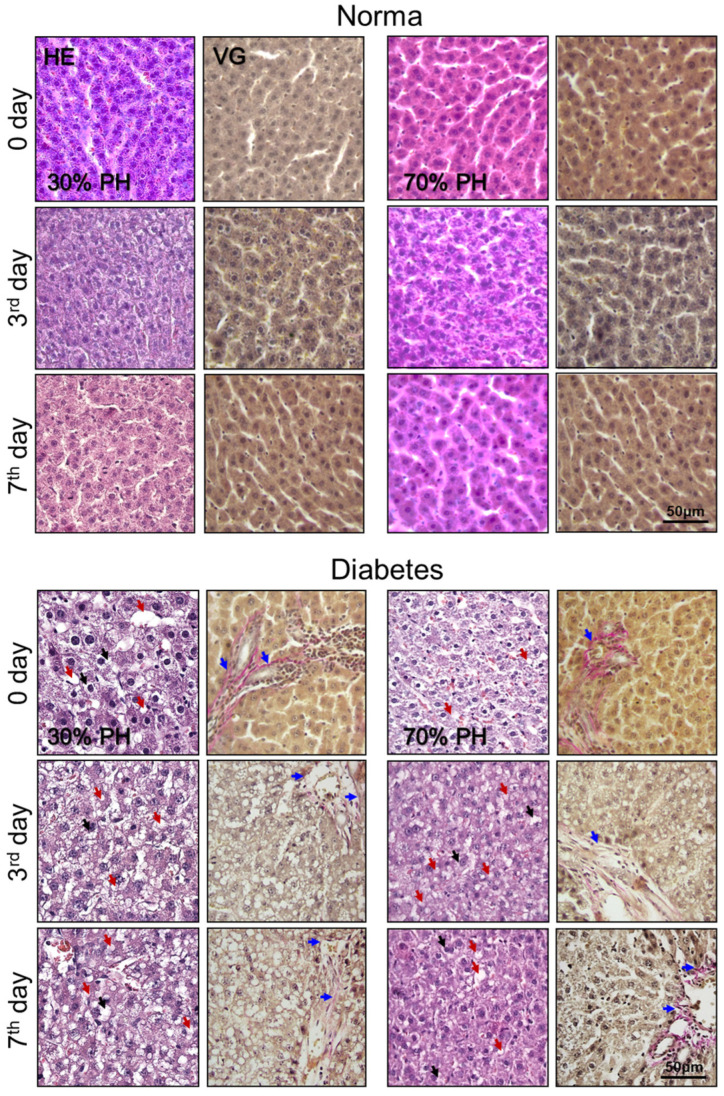
Histological analysis of the liver tissue during normal regeneration and during liver regeneration with induced DM: hematoxylin and eosin (HE) and Van Gieson (VG) staining; red arrows indicate lipid droplets; black arrows indicate vascularized hepatocytes; blue arrows indicate collagen. Normal regeneration—30% PH (*n* = 20, for day 0, and *n* = 10, for the 3rd and 7th days) and 70% PH (*n* = 20, for day 0, and *n* = 10, for the 3rd and 7th days), DM—30% PH (*n* = 12, for day 0, and *n* = 6, for the 3rd and 7th days) and 70% PH (*n* = 12, for day 0, and *n* = 6, for the 3rd and 7th days).

In the induced DM model, histological analysis showed the presence of microvesicular steatosis with the presence of portal fibrosis prior to the induction of regeneration (0 day). Furthermore, by the 3rd and 7th days after both 30% PH and 70% PH, we identified an advanced form of steatosis—NASH, with the development of macrovesicular steatosis (the cytoplasm is replaced by a large single lipid droplet that displaces the nucleus to the edge of the cell). NASH can be diagnosed by the presence of hepatocellular steatosis, lobular inflammation and varying degrees of fibrosis, and in our work, we also observed periportal fibrosis. Other dominant features of NASH are the vacuolation of hepatocytes in the centrizonal area, and varying degrees of hepatocyte necrosis and necro-apoptosis (Figure 6) [7,34]. The results of the histological analysis are consistent with the data of multiphoton microscopy and SHG, which also showed the appearance of areas with the foci of lipid infiltration and collagen accumulation.

### 2.6. Measurements of Liver Weight Recovery

To assess the effectiveness of liver recovery relative to the initial volume at different stages of DM, we determined the weight of the liver before PH, and calculated the subsequent liver-to-body-weight ratios on the 3rd and 7th days after either 30% or 70% PH, i.e., the “absolute weight”. In addition, we analyzed changes in liver weight at different stages of the pathologies during regeneration, relative to normal regeneration, i.e., the “relative weight”. The results are presented in Table 1.

An analysis of the ratio of liver weight to body weight in the rats showed that with the development of DM, there is an increase in liver weight compared to normal regeneration. However, the increase in the liver-to-body-weight ratio of the diabetic liver during regeneration is not associated with a recovery of the functional liver parenchyma, but with the accumulation of lipid droplets in the hepatocytes and an increase in the amount of fibrous tissue. This result is consistent with our earlier results on the development of liver steatosis and fibrosis [18].

### 2.7. Morphometric Analysis

**Figure 7:**
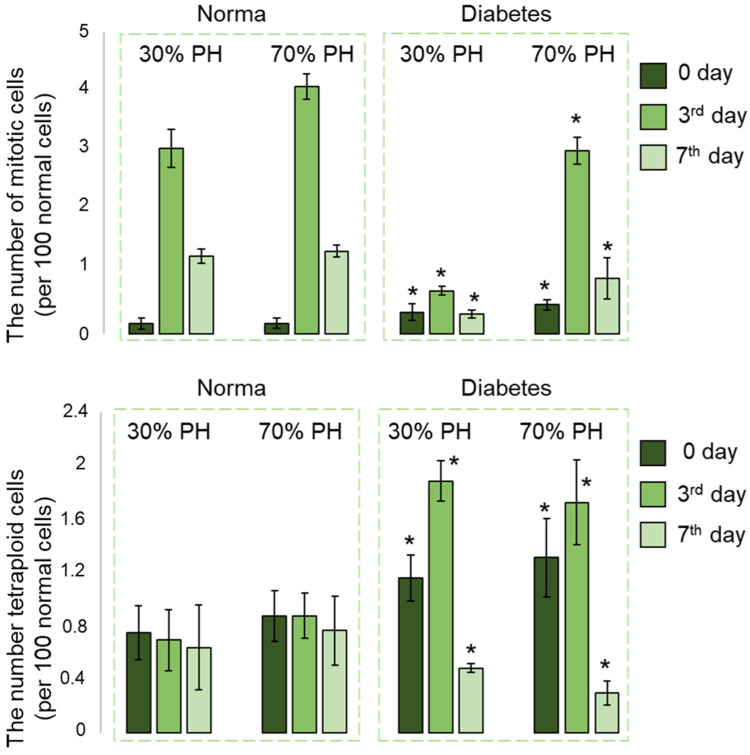
Morphometric analysis of liver tissue during normal liver regeneration and during liver regeneration with induced DM. The charts show the number of tetraploid cells and the number of mitotic cells during regeneration. Values are presented as the number of dividing cells per 100 non-dividing cells. Mean ± SD. *—statistically significant difference compared to the corresponding time point for normal regeneration, *p*-value ≤ 0.05. Normal regeneration—30% PH (*n* = 10, for each time point) and 70% PH (*n* = 10, for each time point), DM—30% PH (*n* = 12, for day 0, and *n* = 6, for the 3rd and 7th days) and 70% PH (*n* = 12, for day 0, and *n* = 6, for the 3rd and 7th days).

Morphometric analysis showed that during normal regeneration, the number of mitotic (dividing) cells had increased sharply by the 3rd day of liver regeneration, followed by a decrease in the number of mitotic cells by the 7th day. At the same time, the proliferative activity of the hepatocytes after 70% PH was higher than after 30% PH. In our model of induced DM after 30% PH, the numbers of mitotic cells on the 3rd and 7th days after PH were significantly lower compared to normal regeneration after 30% PH. By contrast, after 70% PH in the induced DM model, the number of mitotic cells on the 3rd day had increased sharply compared to day 0, although the values still did not reach the level of the corresponding values for normal regeneration after 70% PH. Thus, even despite the reduced regenerative potential of the liver in DM, following large volumes of resection, compensatory proliferation of the hepatocytes was activated (Figure 7).

On day 0, the number of tetraploid cells in the case of induced DM was already significantly higher than the numbers during normal regeneration for both 30% PH and 70% PH. Furthermore, by the 3rd day after PH the number of tetraploid cells in liver with induced DM had increased significantly, although there was subsequently a sharp decrease by the 7th day after PH (Figure 7). The decrease in proliferative activity is also confirmed by molecular analysis data.

## 3. Discussion

In this work, we have identified optical criteria for determining the reduced regenerative potential of liver in induced DM. Standard methods for assessing the presence of hepatic pathology, such as histological analysis of the tissue, are time-consuming and do not allow analysis of fresh tissue samples. Our newly identified imaging criteria for the assessment of the metabolic state of hepatocytes are promising for the express assessment of the regenerative potential of liver tissue before surgery.

The metabolic state of the hepatocytes is a more sensitive marker of pathological processes at the level of individual cells but can also be associated with liver function even at the level of the whole organ [35]. Using our approach based on fluorescence bioimaging methods, it is possible to supplement, or even to substitute, such laborious, time-consuming and less specific methods as histological analysis and biochemical blood tests. Both our group [36,37,38] and other authors have shown that regeneration in healthy livers is characterized by an increase in OXPHOS to meet the energy demands of the dividing hepatocytes. Thus, an increase in OXPHOS is an important criterion indicating successful liver regeneration. However, in diabetic livers, there is a decrease in the intensity of OXPHOS in the hepatocytes, this being primarily associated with the development of mitochondrial dysfunction, accompanied by a decrease in mitochondrial ATP synthesis. In particular, it is known that although DM causes a marked increase in the mitochondrial volume fraction there are highly diminished densities of mitochondrial cristae and damage to the nuclear membranes also occurs [39,40,41]. Mitochondrial oxidative stress further triggers a series of deleterious effects on the mitochondrial respiratory chain by producing reactive oxygen species (ROS) causing DNA damage and mutations [42]. Indeed, in induced DM (before the induction of regeneration), we observed macrovesicular steatosis, which is characteristic of mitochondrial dysfunction. In addition, following the induction of regeneration on the 3rd and 7th days after PH, a more severe condition develops—NASH, which is characterized by even more pronounced mitochondrial dysfunction and significant lipotoxicity [43]. Using bioimaging methods, we found that the development of DM was characterized by the absence of a jump in the values of a2 and a3 on the 3rd day after PH. That is, there was no increase in the intensity of OXPHOS in the proliferating hepatocytes, even though this is necessary for effective regeneration.

In hepatocytes, under hyperglycamic conditions both the glycolytic pathway and the Krebs cycle can be intensively fluxed by glucose [44]. Glucotoxicity refers to the toxic effects on cells and tissues of hyperglycemia and excess carbohydrate intake. It is intrinsically linked to insulin resistance, which facilitates hyperglycemia. Excess carbohydrates can be converted into free fatty acids (FFA) and triglycerides (TGs), and, subsequently, hepatotoxic lipids such as lysophosphatidyl choline, ceramides, and free cholesterol may accumulate [22]. The key issue is not the quantity of liver fat but the types of lipid molecules that are accumulated by the hepatocytes [33]. Adipose tissue insulin resistance in the presence of increased circulating substrates, also promotes TG deposition. Increased plasma levels of TG [30] and low-density lipoproteins plus decreased levels of high-density lipoproteins represent an important risk factor for the development of dyslepidemia and lipotoxicity. FFAs also increase the cellular levels of diacylglycerols, ceramides and long-chain fatty acyl-coenzyme A (CoA), that are lipid metabolites involved in inflammatory processes, mitochondrial dysfunction, increased oxidative stress and in uncoupling OXPHOS, additionally being able to activate a fibrogenic response in hepatic stellate cells that may promote the progression to NASH [39,43,45,46,47].

In addition to a decrease in the intensity of OXPHOS, we revealed the absence of a jump in a3 values on the 3rd day of liver regeneration, which indicates a reduction of the biosynthetic activity of hepatocytes in this pathology, since NADPH is involved in various biosynthetic processes, such as the pentose phosphate pathway, lipogenesis, and nucleic acid synthesis. Also, such a result indicates a decrease in antioxidant defense, due to a decrease in the intensity of the glutathione cycle, in which NADPH is involved, due to a depletion of glutathione, an important component of antioxidant defense [48]. It is likely that the depletion of antioxidant protection is associated with activation of the polyol pathway in hepatocytes. The polyol pathway is usually fairly inactive under euglycemic circumstances but can become a highly active glucose disposal pathway under diabetic hyperglycemic conditions. The major feature of this pathway is its production of NADH, NADPH, and fructose, which play a role in the pathogenesis of DM and its complications [49]. When excess NADH accumulates, the mitochondrial electron transport chain can be overloaded by this electron donor. The direct pressure of this NADH overload would be on complex I, which is a major site for the generation of ROS. Therefore, such oxidants will overwhelm the cellular antioxidant systems, leading to the opening of mitochondrial membrane permeability transition pores that are concurrent with extensive oxidative damage. Excess ROS can also impair the functioning of glutathione reductase, leading to accumulation of the oxidized form of glutathione and further accentuation of the redox imbalance [49]. Furthermore, as glucose flux through the polyol pathway consumes NADPH, it has been suggested that the level of NADPH could be significantly decreased. Additionally, from a chemical point of view, the polyol pathway itself can also compete with glutathione reductase for NADPH [49].

## 4. Materials and Methods

### 4.1. Animal Model

A series of experiments was carried out on male Wistar rats weighing 200–230 g. The animals were divided into 4 groups: normal regeneration—30% PH (*n* = 10, for each time point) and 70% PH (*n* = 10, for each time point), DM—30% PH (*n* = 12, for day 0, and *n* = 6, for the 3rd and 7th days) and 70% PH (*n* = 12, for day 0, and *n* = 6, for the 3rd and 7th days). Before induction of DM, the animals were subjected to 12 h starvation. DM was induced using an intraperitoneal injection of streptozotocin (STZ). Before the procedure, the rats were anesthetized with Zoletil 6 mg/kg (Virbac, Hamilton, New Zealand) and Xylazine 90 mg/kg (Bayer, Leverkusen, Germany). The STZ concentration used was 65 mg/kg of body weight. DM was confirmed by measurements of blood glucose concentrations using a glucometer (AccuChek Active; Roche Diabetes Care, Bern, Switzerland), being diagnosed as present when the blood glucose level increased to 16.7 mmol/L. The induction of DM lasted 20 days. The measurements of blood glucose levels were performed 2 times a day during the entire duration of the experiment. Starting from the 7th day after STZ administration, the rats were injected with insulin NovoMix^®^ 30 FlexPen^®^ (Novo Nordisk, Bagsværd, Denmark) in the morning 10.5 ± 3 U, and in the evening 9.6 ± 3 U, depending on the blood sugar level.

Animals that failed to develop DM were excluded from the study (*n* = 3). The experiment lasted 3 months. Control measurements of blood glucose levels were performed every 2 days during the entire duration of the experiment. Starting from the 7th day after the administration of STZ. Additionally, the successful induction of DM was confirmed by glucose tolerance testing. For this, a 40% glucose solution was injected intravenously at a dose of 0.5 g/kg. After that, at several time points (0, 30, 60 and 120 min), blood was taken for analysis of its glucose level.

The regeneration process was induced by either 70% or 30% partial hepatectomy (PH) [50]. After resection, each animal was placed in a clean cage and kept under the standard conditions of an SPF vivarium. To analyze the state of the liver tissue at different stages of the hepatic pathologies before the induction of liver regeneration, we examined ex vivo liver samples taken during the resection (day 0). On the 3rd and 7th days after PH, animals were removed from the experiment by decapitation, and remnant samples (whole organ) were taken from the animals for study.

### 4.2. Measurements of Liver Weight Recovery and Morphometric Analysis

To determine the effectiveness of liver recovery, the liver weight was measured before the induction of regeneration and at different stages of the recovery process. The initial liver weight was calculated according to the following formula: *weight of resected liver (g)/0.7* for 70% PH, and *weight of resected liver (g)/0.3* for 30% PH. The liver-to-body-weight ratio was calculated according to the following formula: *(weight of the liver remnant (g)/body weight after PH (g))/(initial weight of the liver (g)/body weight before PH (g))*—this value was taken as the “absolute percentage of liver weight recovery”, i.e., the “absolute weight”. To assess the percentage of recovery of liver tissue with induced pathologies relative to the corresponding day of normal regeneration, we calculated the relative percentage of liver weight recovery, i.e., the “relative weight”. For this purpose, the values of “absolute weight” for normal liver regeneration were taken as 100% (absolute weight_norm_) and divided into the “absolute weight” for the liver with pathology (absolute weight_path_) at the corresponding time point of regeneration, i.e., the 3rd or 7th day after PH: *absolute weight_path_(g)/absolute weight_norm_(g)* × *100%*.

The morphometric analysis was carried out on histological sections stained with hematoxylin and eosin and Van Gieson. For each sample, 10 micrographs were obtained (×400) using a Leica DM 2500 microscope (Leica Biosystems, Wetzlar, Germany). The following indicators were evaluated: the number of tetraploid hepatocytes (cells with brightly colored, enlarged nuclei), the number of binucleate cells, and the number of mitotic cells. The average values of all these parameters were calculated in proportion to 100 normal cells [51].

### 4.3. Multiphoton Microscopy

All fluorescence investigations of fresh liver samples were performed using an LSM 880 (Carl Zeiss, Jena, Germany) equipped with a Ti:Sapphire femtosecond laser (Becker & Hickl GmbH, Berlin, Germany) (repetition rate: 80 MHz, pulse duration less than 100 fs) and a time-correlated single photon counting (TCSPC) system (Simple-Tau 152, Becker & Hickl GmbH, Berlin, Germany). The average laser power used was about 10 mW. A C Plan-Apochromat 40×/1.3 oil immersion objective was used to collect the fluorescence signal from 10 fields of view for each sample. Both NAD(P)H fluorescence intensity images and FLIM data were acquired. NAD(P)H: λex = 750 nm, λem = 450–490 nm. Using excitation at a wavelength of 800 nm, we visualized cell autofluorescence (red), detection range 433–660 nm, and the SHG of collagen (green), detection range 371–421 nm. We performed a quantitative assessment of the NAD(P)H autofluorescence intensity in the cytoplasm of the hepatocytes by manual selection of ~40 × 40-pixel zones as regions of interest (ROIs), using ImageJ software (version 1.43u, National Institutes of Health, Bethesda, MD, USA).

The FLIM analysis was performed using SPCImage 8.3 software (Becker & Hickl GmbH, Berlin, Germany) with a tri-exponential decay model. To maintain a minimum of 10,000 counts per pixel, the binning parameter was set at 3. The goodness-of-fit model was assessed using the χ^2^ values, which were in the range of 0.9–1.2 (Figure S3). The following parameters were analyzed in 20–30 regions of the cell cytoplasm for each field of view: tm (ps), the amplitude weighted mean fluorescence lifetimes; t1 (ps) were fixed at 400; t2 (ps), fluorescence lifetimes of the bound form of NADH; t3 (ps), fluorescence lifetimes of NADPH; the relative contributions of the free, a1 (%), and the bound, a2 (%), forms of NADH; and the relative contribution of NADPH, a3 (%).

### 4.4. Real-Time PCR

The isolation of total RNA from the samples was performed according to the protocol for the Quick-Direct-zol DNA/RNA Miniprep kit (#D7001, Zymo Research, Irvine, CA, USA). Before the reverse transcription reaction, the samples were treated with DNAase TURBO DNA-free™ Kit (#AM1907, Invitrogen, Waltham, MA, USA). Real-time PCR was performed on a Bio-Rad CFX96 machine (Bio-Rad, Hercules, CA, USA) using a reaction mixture based on SYBR Green (#7567 Invitrogen, Waltham, MA, USA). The PCR reaction mixture contained 1x GeneAmp PCR Buffer I (#8080129, Applied Biosystems, Waltham, MA, USA), 250 µM of each dNTP, 0.5 nM of each primer, and 1 U of Taq M polymerase (#751-100, Intifica, Saint Petersburg, Russia); the total concentration of Mg^2+^ in the reaction was 3 mM, the reaction volume was 20 µL. The temperature profile of cycles: (1) 95 °C for 10 min (enzyme activation step); (2) 40 cycles of 95 °C for 15 s, 60 °C for 30 s, and 72 °C for 30 s; (3) hybridization 1 min 95 °C and 1 min 40 °C; (4) melt curve analysis with measurements between 60 °C and 95 °C. The reaction efficiency was calculated using LinRegPCR app. The stability of putative reference genes (Hprt, Ipo8, Ywhaz, Gusb, B2m) was analyzed using an algorithm GeNorm integrated into qBase 3.4 software. The following reference genes were used for expression normalization: Hprt, B2m (GeNorm V < 0.15). Quantitative RT-PCR analysis was performed using the CFX Maestro 2.3 software. The primer sequences for RT-PCR are presented in Table S2.

### 4.5. Histological Analysis

For histological studies, the liver ex vivo samples were fixed in a 4% solution of paraformaldehyde, passed through isopropyl alcohol, and embedded in paraffin. Deparaffinized 7 µm sections were stained with hematoxylin, eosin, and Van Gieson’s method according to the standard protocol [52]. For each sample, ten microimages (×40) were obtained using a Leica DM 2500 microscope (Leica Biosystems, Wetzlar, Germany). Afterwards, a standard morphological analysis of the liver tissue structure was performed, evaluating the presence of any dystrophic changes of the tissue.

### 4.6. Statistics

To determine the statistical significance of changes in the degree of tissue regenerative capacity of the liver with induced pathology, the nonparametric Mann–Whitney U test was used. Comparative analyses were performed between the recovered weights of livers with induced pathology on the 3rd and 7th days after 30% PH or 70% PH with the corresponding time point of normal liver regeneration; *p*-value ≤ 0.05.

For each time point of the experiment, we obtained 8–10 NAD(P)H autofluorescence and FLIM images. On every image, we performed a quantitative assessment of the NAD(P)H autofluorescence intensity in the cytoplasm of 30 hepatocytes (in zones with a high NAD(P)H autofluorescence intensity) and for 10–30 hepatocytes (in zones with a low NAD(P)H autofluorescence intensity), excluding the nucleus. For each image, we also determined the FLIM parameters in the cytoplasm of 30 hepatocytes (in zones with a high NAD(P)H autofluorescence intensity). R-language was used for statistical calculations. Statistical differences between the different groups were analyzed using the pairwise multiple comparison procedure. After the tests for normality (Shapiro–Wilkes) and equal variance (F-test) showed a normal distribution of data, the pairwise t-test method was used, for pairwise comparison, using the Bonferroni correction; *p*-value ≤ 0.05.

Quantitative RT-PCR analysis was performed using CFX Maestro 2.3 software. For statistical data processing, log2 transformed expression data was extracted from CFX Maestro 2.3 software, then two-way ANOVA, the Dunnett test, and independent *t*-tests were carried out using JASP (Version 0.18.1). The analysis of changes in gene expression was carried out using two-way ANOVA, where two factors were specified: “day” after resection (0, 3rd and 7th), and “pathology” (presence/absence of DM). To compare changes in gene expression on the 3rd and 7th day after PH with day 0 (before PH) (for normal regeneration and for regeneration with concomitant DM), we used a one-way ANOVA with the Dunnett test. An independent t-test was used to compare gene expression at corresponding time points for DM and normal regeneration.

## 5. Conclusions

Using an approach based on label-free methods of multiphoton microscopy, SHG and FLIM, we identified criteria for indicating reduced liver regenerative potential in the presence of induced diabetes mellitus type 1. These include the appearance of zones with a reduced NAD(P)H autofluorescence signal associated with the lipid infiltration of hepatocytes and the foci of fibrosis. In addition, there was a decrease in the fluorescence lifetime contributions of the bound form of NADH and NADPH and the absence of any expected jump in the values of these parameters during liver regeneration. Such results show the depletion of energy resources in the pathological liver, which significantly reduces the efficiency of liver restoration. Determining the state of these criteria will expand the possibilities for assessing the regenerative potential of a liver remnant when considering the presence of various pathologies.

## Figures and Tables

**Table 1:** Analysis of the recovery of liver weight.

	Normal Regeneration				Diabetes Mellitus			
	30% PH		70% PH		30% PH		70% PH	
	3rd Day	7th Day	3rd Day	7th Day	3rd Day	7th Day	3rd Day	7th Day
Absolute weight (%)	73.4	88.4	80.9	92.9	97.8 *	93.5 *	83.8	96.6
Relative weight (%)	-	-	-	-	133.3	105.7	103.7	103.9

*—significant difference from the corresponding time point of normal regeneration.

## Data Availability

Data available on request from the corresponding author.

## Supplementary Materials

The following supporting information can be downloaded at: https://www.mdpi.com/article/10.3390/ijms242317016/s1.
