# Supplementary material for: The Effect of Diabetes Mellitus Type 1 on the Energy Metabolism of Hepatocytes: Multiphoton Microscopy and Fluorescence Lifetime Imaging
Source: Int J Mol Sci. 2023 Nov 30;24(23):17016. doi: 10.3390/ijms242317016 (PMC10706954; doi:10.3390/ijms242317016)
Supplement: Supplementary file 1 [file ijms-24-17016-s001.zip › ijms-2708903-supplementary.pdf]

Supplementary Materials

The Effect of Diabetes Mellitus Type 1 on the Energy Metabolism of Hepatocytes: Multiphoton Microscopy and Fluorescence Lifetime Imaging

Svetlana Rodimova [1], Nikolai Bobrov [2], Artem Mozherov [1,2], Vadim Elagin [1], Maria Karabut [1], Polina Ermakova [1], Ilya Shchechkin [1,3], Dmitry Kozlov [1,3], Dmitry Krylov [1,3], Alena Gavrina [1,3], Aleksandra Kashina [1], Vladimir Zagainov [2,4], Elena Zagaynova [1,5] and Daria Kuznetsova [1,3,*]

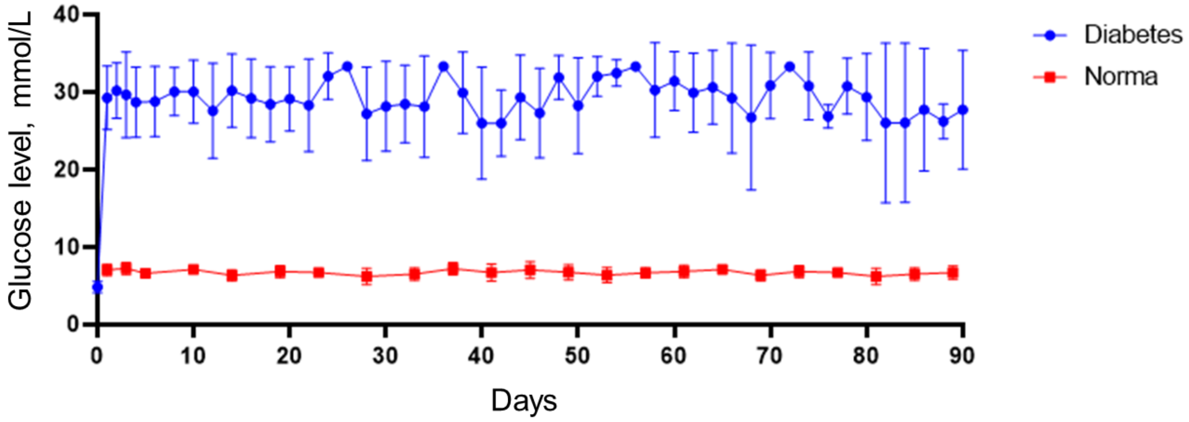

Figure S1. Dynamics of changes in glucose levels in rats during the development of diabetes mellitus type 1 following 30% or 70% PH.

Table S1. Fluorescence lifetime values of the bound form of NADH (t2) and NADPH (t3) in hepatocytes.

30 % PH	Normal regeneration			Diabetes Mellitus		
	t2, ps	t3, ps	tm, ps	t2, ps	t3, ps	tm, ps
0 day	2276±101	4105±317	1276±39	1846±216*	5035±370*	1035±229*
3rd day	2194±113	4130±421	1387±43	1975±179	3960±371	1071±219*
7th day	2094±96	3861±276	1312±55	1956±171	4033±297	1038±185*

70 % PH	Normal regeneration			Diabetes Mellitus		
	t2, ps	t3, ps	tm, ps	t2, ps	t3, ps	tm, ps
0 day	2276±101	4105±317	1276±39	1699±228*	4895±381*	1002±110*
3rd day	2194±113	4130±421	1387±43	1683±223*	4999±356*	1160±23*
7th day	2094±96	3861±276	1312±55	1639±184*	5349±211*	1110±20*

*—statistically significant differences compared to the corresponding time point for normal re-generation

Table S2. The primer sequences for RT-PCR.

Primer Target	Primer Sequence
CYP2E1	F: AGGCTGTCAAGGAGGTGCTACT<br>R: AAAACCTCCGCACGTCCTTCCA
SREBPf1	F: GCTCACAAAAGCAAATCACT<br>R: GCGTTTCTACCACTTCAGG
Acaca	F: GCGGCTCTGGAGGTATATGT<br>R: TTAGCGTGGGGATGTTCCCT
SIRT1	F: GGATCATTCAGTGTCATGGTTC<br>R: AGGACACCGAGGAACTACCTG
HGF	F: TTGCCCTATTTCCCGTTGTGA<br>R: ACCATCCACCCTACTGTTGT
UCP2	F: TCTCCCAATGTTGCCCGAAA<br>R: CAAGGGAGGTCGTCTGTCAT
Mlxipl	F: AATGGGATGGTGTCTACCGC<br>R: GAATTCAGGACAGTTGGCCG
TNFR1	F: CCAAGTGCCACAAAGGAACC<br>R: CCGACATGTCTTGCAACTGAG
PPARa	F: GACTAGCAACAATCCGCCTTT<br>R: GCCTCCTTGTTTTCAACGCC
CYP7A1	F: CTGCCGGTACTAGACAGCATC<br>R: CCGTCCTCAAGATGGAGAGTG
CAT	F: GCTCCGCAATCCTACACCAT<br>R: GGACATCGGGTTTCTGAGGG
SREBPf2	F: CGCCCTTCAAGTACCAACCC<br>R: CTCCTTCTTTGGGAGGCTCG
Nr1h3	F: CGACTTCGAGTCACGCCTTG<br>R: AGGGAGCTGTTTGGCAAAGT
il6R	F: AAGCAGGTCCAGCCACAATGTAG<br>R: CCAACTGACTTTGAGCCAACGAG
HPRT	F: CTCATGGACTGATTATGGACAGGAC<br>R: GCAGGTCAGCAAAGAACTTATAGCC
IPO8	F: GTGGAGTTCCCTGTGCGTC<br>R: ACGAATCTGCTGCCGGTCAT
Gusb	F: CCGTGGAACAGGGAATGAG<br>R: CTCAGGTGTTGTCATCGTCA
B2M	F: CGAGACCGATGTATATGCTTGC<br>R: GTCCAGATGATTCAGAGCTCCA
Ywhaz	F: GATGAAGCCATTGCTGAACTTG<br>R: GTCTCCTTGGGTATCCGATGTC

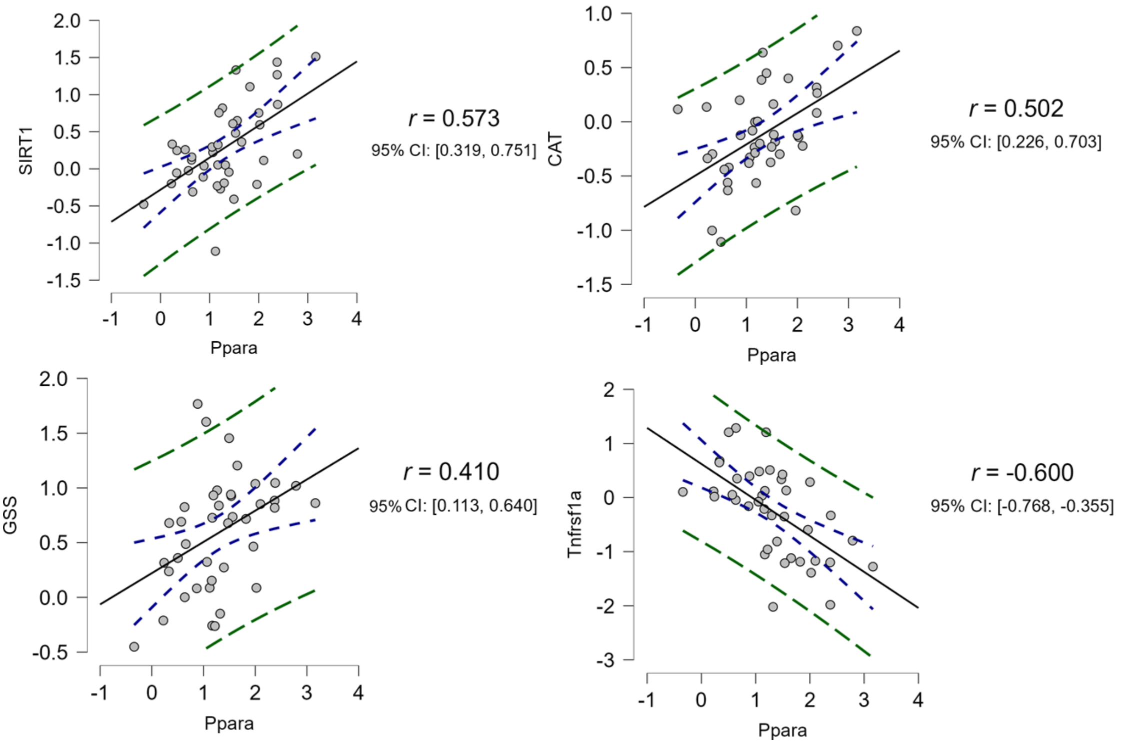

Figure S2. Scatter plot of correlation of genes expression with confidence interval and trend line, Pearson's r and 95% confidence interval.

The goodness of fit model assessment

Goodness of fit model was evaluated on the basis of $\chi 2$ values, which were in the range of 0.9–1.2. Figures showing the typical tri-exponential fitting and fluorescence decay of NAD(P)H.

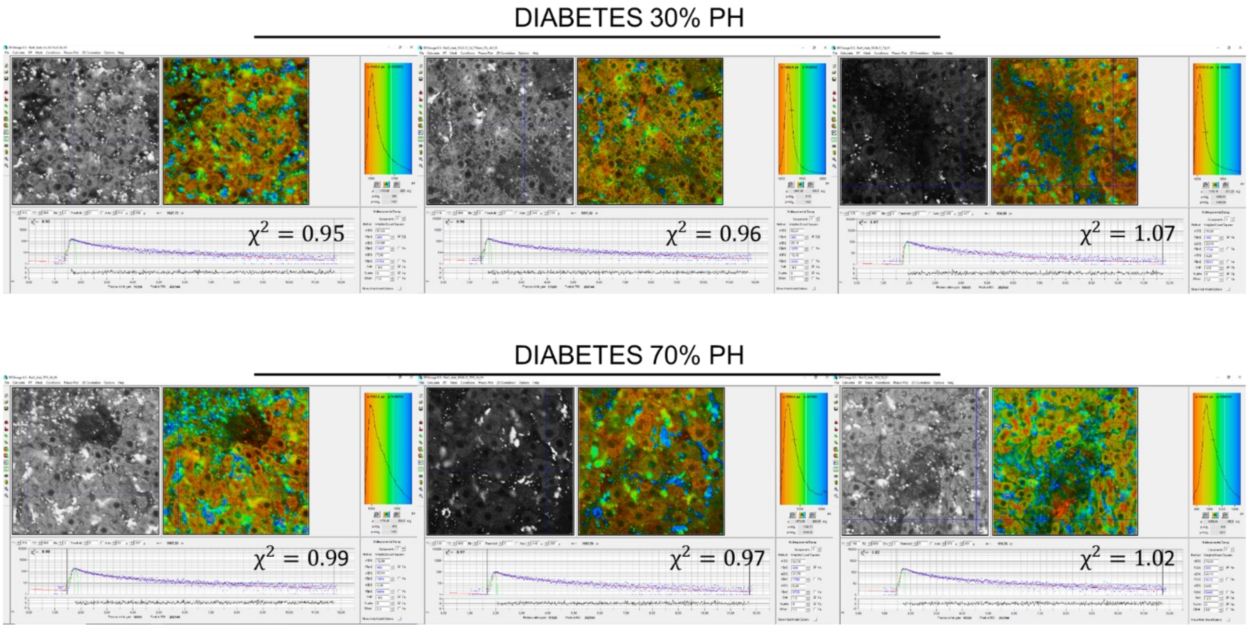

Figure S3. Image of typical tri-exponential fitting and fluorescence decay of NAD(P)H. The images were obtained using SPCImage software. The values of the goodness of the fit have been enlarged and placed on the right side of each image.
